# An ELISA assay using a combination of recombinant proteins from multiple strains of *Orientia tsutsugamushi* offers an accurate diagnosis for scrub typhus

**DOI:** 10.1186/s12879-017-2512-8

**Published:** 2017-06-10

**Authors:** Chien-Chung Chao, Zhiwen Zhang, Tatyana Belinskaya, Wilawan Thipmontree, Wiwit Tantibhedyangkul, Saowaluk Silpasakorn, Ekkarat Wongsawat, Yupin Suputtamongkol, Wei-Mei Ching

**Affiliations:** 1 Viral and Rickettsial Diseases Department, Infectious Diseases Directorate, Naval Medical Research Center, Silver Spring, MD USA; 2 Department of Preventive Medicine and Biostatistics, Uniformed Services University of the Health Sciences, Bethesda, MD USA; 3 Department of Medicine, Maharat Nakhon Ratchasima Hospital, Nakhon Ratchasima, Thailand; 4 Department of Medicine, Faculty of Medicine, Siriraj Hospital, Mahidol University, Bangkok, Thailand; 5 Department of Immunology, Faculty of Medicine, Siriraj Hospital, Mahidol University, Bangkok, Thailand

**Keywords:** Scrub typhus, Diagnosis, Elisa, Recombinant protein antigens

## Abstract

**Background:**

Scrub typhus (ST) is a disease caused by an obligate intracellular bacterium, *Orientia tsutsugamushi*, an organism that requires a BSL3 laboratory for propagation. The disease is hallmarked by an eschar at the site of the chigger bite, followed by the development of fever, malaise, myalgia, anorexia, and papulomacular rash. Indirect immunofluorescent assay (IFA) is the gold standard for scrub typhus diagnosis, however, the subjectivity of the assay, the need for a specialized laboratory and instruments has limited the wide use of the test in resource limited areas.

**Methods:**

A recombinant-protein based enzyme linked immunosorbent assay (ELISA) using the most abundant and immunodominant protein for the detection of *Orientia* specific antibodies in serum has been developed. The performance of the assay was evaluated using prospectively collected acute sera from 248 randomly selected patients in Thailand. The ELISA assay was evaluated using two different cutoff values.

**Results:**

The receiver operating characteristic (ROC) curve generated cutoff values gave slightly better consistency with diagnosis of ST than those cutoff values established by averaging ELISA optical density of known negatives at 99% confidence interval. Both cutoff values provided similar statistical parameters when compared with the diagnosis of ST, indicating the validity of both calculations to derive cutoff values. These results suggest that both IgG and IgM ELISA performed well to accurately diagnose scrub typhus cases in endemic areas using only acute serum samples.

**Conclusions:**

We have successfully developed an ELISA assay for the detection of *Orientia*-specific antibodies in serum that could provide effective screening of acute sera under clinical setup and it is also a useful assay to estimate seroprevalence in various endemic areas.

**Electronic supplementary material:**

The online version of this article (doi:10.1186/s12879-017-2512-8) contains supplementary material, which is available to authorized users.

## Background

Early detection, accurate diagnosis, and timely treatment of rickettsial disease are imperative to improve disease outcome during outbreaks. Scrub typhus (ST) is one of the most common rickettsial diseases caused by the infection of *Orientia tsutsugamushi,* an obligate intracellular Gram-negative bacterium [1, 2]. The disease is characterized by fever, rash, eschar, pneumonitis, meningitis, and in some cases, disseminated intravascular coagulation that may lead to circulatory failure [3]. It can cause up to 70% mortality with a median mortality of 6.0% [4]. Reports from India documented 12–17% case fatality rate in recent years [5, 6]. In 2012, the deadly disease spread to 10 different districts and more than 140 cases were reported. At least 14 people died of the disease [7, 8]. In 2015, a large scale scrub typhus epidemic (126 cases) with high case fatality rate occurred in Nepal and it is still on going in 2016 with additional fatal cases reported [9, 10]. In India, a total of 700 cases with 20 death have been reported in 2016 [10]. In the past, scrub typhus cases usually were reported primarily from the rural populace, but recently it is being increasingly detected in people residing in expanding cities [11].

Clinical manifestations of scrub typhus vary widely from a mild and self-limiting febrile illness to a more severe illness that may be fatal due to multi-organ failures [7, 12]. Traditionally, the diagnosis of scrub typhus mainly relies on serologic tests. When both acute and convalescent sera are available, the disease could be diagnosed retrospectively in cases of seroconversion or a > 4-fold rise in antibody titers between the two sera [13]. Currently, laboratory diagnosis of the disease employs immunological techniques such as the Weil-Felix (WF) test, passive hemagglutination assay, indirect immunofluorescence (IFA) test, indirect immunoperoxidase (IIP) test, and enzyme linked immunosorbent assay (ELISA) [14]. WF test based on the agglutination of OXK strain *Proteus mirabilis* is the simplest detection method, which has been widely used for clinical identification at hospitals in tropical countries, especially in Thailand [15]. However, it is neither specific nor sensitive. IFA and IIP tests have high sensitivity and specificity, but can be troublesome for inexperienced technicians because of microscopic evaluation [16]. A set of algorithms for diagnosing ST, Scrub Typhus Improved Criteria (STIC) has suggested the use of isolation of *O. tsutsugamushi*, admission of IFA IgM titer >1:12,800, or 4-fold titer increase in IFA IgM in paired sera or positive PCR results [17] targeting two of the three genes (56 kDa, 47 kDa or groEL). Recent work from the same group led by Lim et al. [18, 19] has determined the optimal IFA cutoff titer by latent class analysis (LCA) to offer an additional tool for the accurate diagnosis of ST. However, it is difficult to implement the same STIC in laboratories across different geographic areas to accurately diagnose ST since 1) isolation of *O. tsutsugamushi* and routine IFA require a BSL3 laboratory and experienced staff, 2) it is also difficult to establish an IFA cutoff titer using the complicated LCA algorithm which requires specially trained experts.

The recombinant protein of the most immunodominant and abundant 56 kDa protein antigen from the Karp strain (rKp56) of *O. tsutsugamushi* was first cloned in 1998 [20] and was used to produce a rapid flow assay [21] for the diagnosis of scrub typhus. This recombinant protein antigen was used in ELISA for laboratory diagnosis of scrub typhus in two outbreaks in Camp Fuji in 2000 and 2001 [22] and was used as a potential vaccine candidate in a mouse and monkey model [23, 24]. Development of 56 kDa recombinant proteins from additional Orientia strains has resulted in the selection of three recombinant proteins that are reactive to 18 disparate isolates of *Orientia* that encompasses over 85% of isolates [25], suggesting that the combination of these three recombinant proteins could offer sensitivity and specificity better than the previously used assay that was based on a single rKp56. In fact, a rapid test was constructed based on the combination of these three recombinant proteins by InBios and was recently evaluated by 4 different laboratories demonstrating a sensitivity and specificity for IgG or IgM at greater than 85% in comparison to IFA using the optimal cutoff established by each group [12, 26–28]. These results suggest the combination of these recombinant protein antigens provided reliable and broadly reactive tests. Furthermore, InBios has developed an IgM ELISA kit using the same recombinant proteins, which has recently been evaluated and shown good performance [29] indicating the potential use of IgM ELISA for diagnosis in endemic areas. Rodkvamtook et al. [30] developed a modified Dot-ELISA using these recombinant proteins to diagnose ST. The performance of this assay for acute and convalescent sera was as good as IFA.

The purpose of this study is to compare and evaluate the performance of an ELISA using a combination of recombinant antigens using prospective collected samples that were diagnosed as scrub typhus using the combined results of IFA and PCR. The focus was to determine whether the sensitivity and specificity of this ELISA is comparable to clinical laboratory confirmed cases using a single acute serum from randomly selected individual fever patients. Our data suggests that the ELISA assay is a potential serological assay for the accurate diagnosis of scrub typhus in endemic areas and is a more quantitative and high-throughput alternative to the IFA.

## Methods

### Patient samples

A total of 800 patient samples were prospectively collected in Thailand under human use protocol COA Si204/2011 between Sep 2010 and Sep 2013. The inclusion criteria were adult >18 years of age with acute fever >38.5 °C for at least 3 days. Patients were excluded from the protocol if they were diagnosed with malaria. There was no identifiable source of infection among these 800 patients and it was suspected that the fever could be due to rickettsial infections. The study subjects were informed that their samples would be stored for future use to detect the presence of pathogens. The evaluation of these de-identified clinical samples by ELISA was approved by the IRB of the Naval Medical Research Center (PJT-15-15). Among these 800 patients, acute sera from 248 patients were randomly selected for this study and they were diagnosed in Siriraj Hospital. The criteria used for diagnosis of various diseases and the distribution of these diseases are shown in Table 1. The prevalence of scrub typhus and murine typhus in these samples was around 15% and 5%, respectively.

**Table 1:** Description of clinical samples included in the ELISA evaluation^a^

Diagnosis	# of samples	Case definition (# of samples)
Scrub typhus^b^	78	IgG or IgM or both ≥400 (61), or 4-fold increase in IgG or IgM titer between convalescent and acute specimens (6), or PCR positive only^d^ (5), or PCR positive and 4-fold increase in titer (6)
Dengue	11	NS1, or IgG or IgM rapid test positive
Murine typhus	2	single IFA IgG and/or IgM titer ≥400, or a 4-fold increase in IgG or IgM titer
leptospirosis	16	single IFA IgG or IgM titer ≥400, or a 4-fold increase in titer of IgG or IgM, or positive PCR for LipL32
Other bacteremia	3	Bacteria culture positive
Co-infection (Scrub typhus and others)^c^	8	IgG or IgM ≥ 400 (7), 4-fold increase in IgG and IgM titer between convalescent and acute specimens (1)
unknown	130	Negative by all above

^a^Samples were determined for the cause of infection using the criteria listed

^b^Five different criteria were applied to determine scrub typhus positive. A sample is considered scrub typhus positive if it is positive by any of the 5 criteria. Numbers in parentheses represent number of cases defined by respective criterion

^c^The same criteria to identify scrub typhus positive were applied to co-infection samples

^d^The 47 kDa or 56 kDa gene was used as targets for PCR

### Recombinant protein antigens

Three recombinant protein antigens were derived from four prototype strains of Karp and TA763 (r56C1), Kato (r56Kt), and Gilliam (r56Gm) *Orientia* [25]. The cloning, expression, purification, refolding and seroreactivity were described previously [25].

### ELISA experiment

The plates were coated with 0.3 μg/well/100 μL of each of the three recombinant proteins at equal amounts overnight at 4C. The experiment was performed as described before [25]. Briefly, the coated plates were first rinsed three times with 1× phosphate buffered saline (PBS) containing 0.1% Tween 20 (1× PBST), blocked with 200 μL/well of 10% milk in 1× PBST and incubated for 1 h (h) at room temperature. Primary antibody (individual serum) was diluted 1:100 in 5% milk in 1× PBST, added to the plate, and followed by 1 h incubation. After washing the plates 3 times with PBST, they were incubated with HRP conjugated goat anti-mouse IgG or HRP conjugated rabbit anti-human IgG (Santa Cruz) at 1:4000 dilution for 1 h at room temperature. At the end of incubation, plates were washed and substrate (Kirkegaard & Perry Laboratories, Gaithersburg, MD) was added. The plates were incubated at room temperature for 15–30 min in the dark and read at 405 nm–650 nm on an UVmax kinetic microplate reader (Molecular Devices, Sunnyvale, CA). The optical density (OD) was used to calculate cutoff values according to Frey et al. [31] or to construct a ROC curve as described below.

### Indirect Immunofluorescent (IFA) assay

The IFA was performed on single acute sera or on acute and convalescent paired sera using an established method [32]. Briefly, pooled antigens of *O. tsutsugamushi* strain Karp, Kato, and Gilliam at equal ratio were spotted on a glass slide. Patient sera were serially diluted two-fold from 1:50 to 1:6400 in PBS containing 2% (*w*/*v*) skim milk, incubated in a humidified chamber for 30 min at 37 °C, and washed three times in PBS. Anti-human IgM and IgG fluorescein isothiocyanate conjugate (Jackson Immuno Research Laboratories, West Grove, PA), diluted in PBS–skim milk diluent containing 0.00125% (*w*/*v*) Evans Blue for counter stain, and incubated as described previously. After incubation, slides were examined by fluorescence microscope (BX50; Olympus, Tokyo, Japan) at a magnification of 400. The endpoint titer was determined as the highest titer that showed fluorescence signal above the background. Known positive and negative control sera were included in each experiment.

### Data analysis

**Determination of ELISA cutoff value to define positive or negative ELISA results.** Two different calculations were performed to determine the best cutoff values for IgG or IgM ELISA.

A) The first cutoff values were determined using the mean of OD and standard deviation of a total of 12 negative controls from endemic areas at 99% confidence interval as described by Frey et al. [31]. The resulted IgG and IgM cutoffs were 0.816 and 0.320, respectively.

B) The second cutoff values were determined by generating a ROC curve using GraphPad Prism v 5.03. This resulted in a table displaying every possible cutoff value for a positive OD. The area under the ROC curve (AUC) was also calculated. The cutoff values of the IgG and IgM ELISA assays were selected as the ones that maximized the sum of sensitivity and specificity by using Youden’s index (*J* = sensitivity + specificity - 1) [33].

**Definition of patient as scrub typhus positive.** A patient is defined as scrub typhus positive if any of the three criteria listed in Table 2 is met. A patient is defined as scrub typhus negative if none of the 3 criteria is met. This definition is to be used to define true positive and true negative to examine the concordance of the ELISA assay with scrub typhus positive or negative diagnosis based on the two aforementioned cutoff calculations. Therefore, if a patient has an IgM or IgG ELISA OD greater than the calculated cutoff values and the patient meets the criteria as scrub typhus positive, then the IgM or IgG ELISA is a true positive.

**Table 2:** List of different criteria to determine the performance of ELISA for diagnosis of scrub typhus

Criteria used to determine ST cases^a^	Patient is considered ST	If ELISA IgG or IgM OD is^b^	
		≥ cutoff	< cutoff
1. IgG or IgM or both ≥400 or 2. 4-fold increase in IgG or IgM titer; or 3. PCR positive	Positive	TP	FN
Otherwise	Negative	FP	TN

^a^If IFA or PCR test results meet any of the criteria, the patient is considered ST positive

^b^The ODs of IgG or IgM ELISA were compared with calculated IgG and IgM ELISA cutoff values, respectively. If it was ≥ cutoff, the ELISA is considered a true positive (TP). If it was < cutoff, the ELISA is considered a false negative (FN)

**Evaluation of the performance of ELISA using cutoff values and scrub typhus positive.** To evaluate the performance of IgG or IgM ELISA, each of the two cutoff values obtained as described were analyzed using the defined scrub typhus positive. The typical 2 × 2 table was constructed for each comparison to calculate sensitivity, specificity, positive predict value, negative predict value, and accuracy. We also calculated the likelihood ratio for a positive test (LR+) and the likelihood ratio for a negative test (LR-). The likelihood ratio was determined by using the following formulas: LR+ = sensitivity/1 - specificity, LR- = 1-sensitivity/specificity. Statistical analysis was performed using GrpahPad Prism v. 5.03

## Results

### Distribution of IFA titer by acute sera of scrub typhus positive patients

**Fig. 1:**
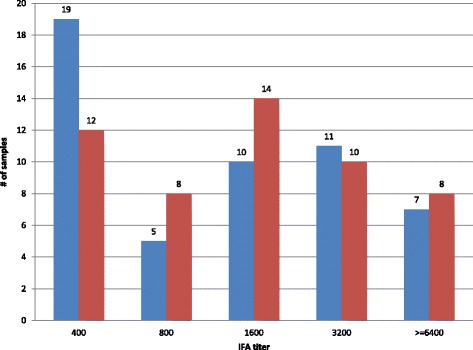
IgG or IgM titer distribution of IFA positive samples. The distribution of IFA titer in the 248 samples used in the ELISA assay. The reciprocal IgG or IgM IFA titer needs to be > = 400 in order to be considered as positive cases of scrub typhus. Blue bars represent number of samples with given IgG IFA titers and red bars represent number of samples with given IgM IFA titers. The numbers given on top of each bar indicate the number of samples having respective IFA titer

While the general prevalence rate of ST is around 15%, our random selection included 86 (34.7%) patients diagnosed with ST according to the criteria listed in Table 2. Among these 86 patients, a total of 66 (76.7%) ST patients were confirmed by IFA titer of acute sera. These 66 patients included 47 IgG IFA positives, 48 IgM positives and 36 positives for both IgG and IgM (Table 3). There were additional samples diagnosed as co-infection of scrub typhus and another disease. Among these co-infections, five were IgG positives, four were IgM positives and two were both IgG and IgM positives (Table 3). The data in Fig. 1 showed the distribution of positive IFA titer of these acute sera.

**Table 3:** Diagnosis of scrub typhus by acute-phase IgG or IgM IFA only^a^

Diagnosis	IgG ≥ 400	IgM ≥ 400	IgG and IgM both ≥400
Scrub typhus	47	48	36
Co-infection	5	4	2

^a^A total of 66 positive samples were identified based on titer of 400 for IgG or IgM or both as determined by IFA

### Diagnosis of scrub typhus by IFA titer and PCR

In the total of 86 positive patients, 66 were determined positive by IFA titer of acute sera. The remaining 20 ST patients were confirmed by PCR positive for 47 kDa or 56 kDa gene or by 4-fold increase of IFA titers between acute and convalescent sera. These included six patients with 4-fold titer increase of IgG or IgM between acute and convalescent sera, two patients with IgG or IgM titer ≥400 in convalescent sera, 5 patients showed PCR positive for 56 kDa and/or 47 kDa gene, 6 patients were positive by both PCR and 4-fold increase of IFA titer and one co-infected patient diagnosed as scrub typhus positive by 4-fold IgG and IgM titer increase (Table 1).

### Correlation between IFA titer and ELISA in acute sera

**Fig. 2:**
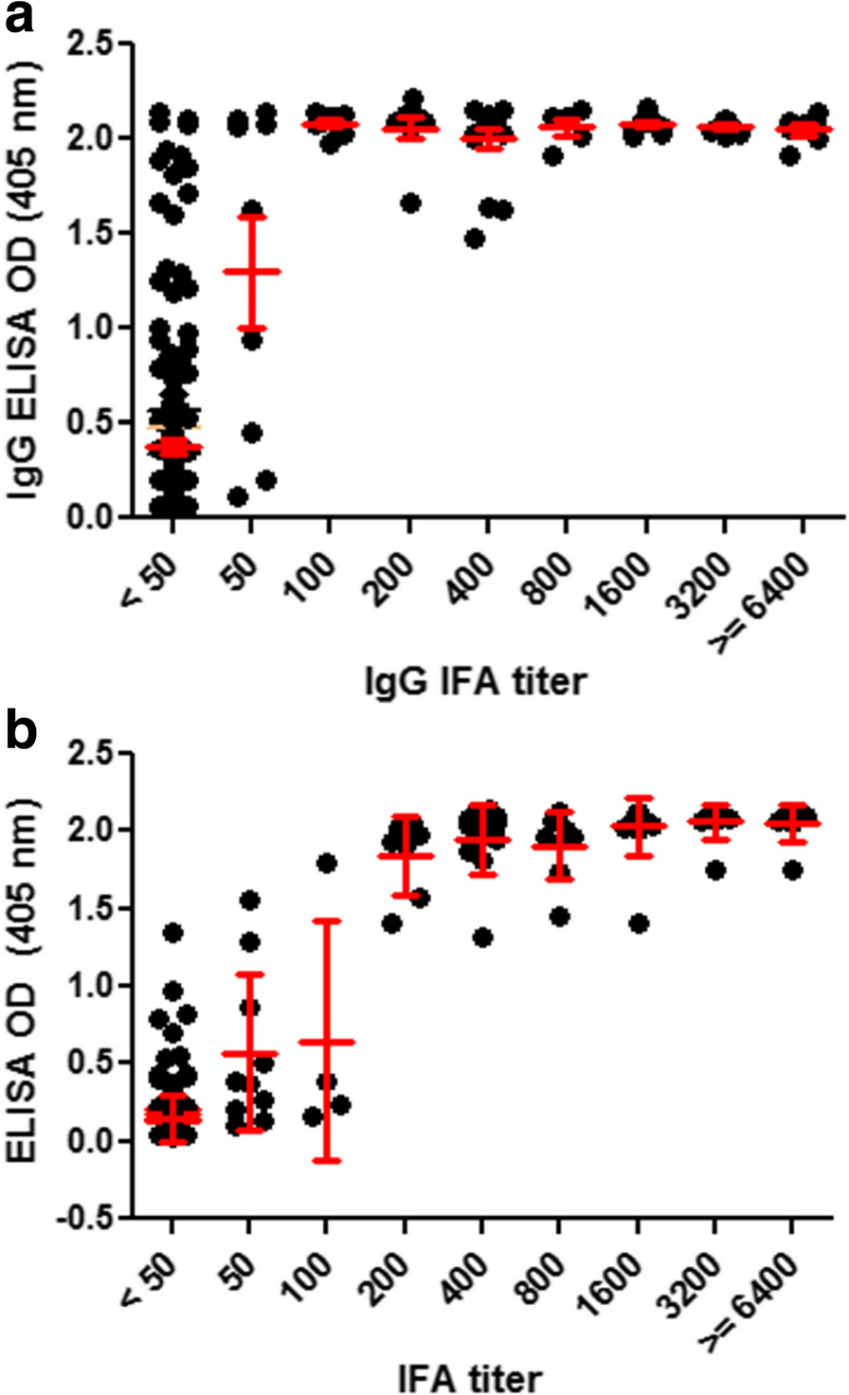
Correlation of IgG (Panel **a**) and IgM (panel B) ELISA OD with IFA titers. The ELISA OD of each sample was plotted against its corresponding titer. Panel **a** shows the correlation of IgG ELISA OD and IFA titers. The correlation co-efficient for IgG was 0.478 for Pearson and was 0.729 for Spearman (*p* < 0.0001). Panel **b** shows the correlation of IgM ELISA OD and IFA titers. The correlation co-efficient for IgM was 0.650 for Pearson and was 0.765 for Spearman (*p* < 0.0001). The mean and standard deviation of ELISA OD of all samples within a given IFA titer is was plotted in red

The ELISA OD correlated well with IFA titer for both IgG and IgM as shown in Fig. 2, Panel a and b, respectively. The correlation co-efficient for IgG was 0.478 for Pearson and was 0.729 for Spearman (*p* < 0.0001). Similarly, the correlation co-efficient for IgM was 0.650 for Pearson and was 0.765 for Spearman (*p* < 0.0001). As shown in Additional file 1: Table S1, all sera with IFA titer ≥400 were ELISA positive using cutoff values of 0.816 and 0.320 for IgG and IgM, respectively. For samples with IFA titer <400, the OD of ELISA IgG correlated well with IFA IgG, as all titer ≥100 were positive by ELISA. Similarly, the OD of ELISA IgM correlated well with IFA IgM for titers ≥200. The correlation between positive IFA titer of single serum with IgG or IgM ELISA OD was shown in Additional file 1: Figure S1.

### Evaluation of the performance of ELISA using cutoff values based on known negative controls at 99% confidence interval

The ELISA cutoff values were 0.816 and 0.320, for IgG and IgM, respectively, determined as described in the Method section. Using these cutoff values, we identified a total of 102 IgG positives and 82 IgM positives with 72 both IgG and IgM positives, resulting in a total of 112 positive patients. All acute sera that were IFA positives were also ELISA positives including those patients with co-infection. In all 86 ST positives, 81 were ELISA IgG positive. Among the 5 acute sera with negative ELISA, one was from a patient who had 4-fold IFA titer increase between acute and convalescent sera, one patient was PCR positive in acute serum and 4-fold titer increase, one was PCR positive in acute serum only, one was positive because the IgG or IgM titer was ≥400 in convalescent serum, and one patient who was diagnosed as ST positive based on a 4-fold titer increase also showed 4-fold increase in IFA titer for leptospirosis, suggesting that it is a co-infection of ST and leptospirosis. For all ELISA positives, 30 were not diagnosed as ST patients. Twenty-three of these were diagnosed as unknown and 18 of them did not have convalescent sera while 5 of them had convalescent sera with negative IFA results. Interestingly enough, 6 ELISA IgG or IgM positives were determined as leptospirosis positive. Among these 6 patients, 2 patients did not have convalescent sera. The other 4 patients still showed ST IFA negative using convalescent sera. One murine typhus patient was ST ELISA positive but IFA IgG/IgM in convalescent serum stayed negative (Additional file 1: Table S2). The performance of the ELISA using these calculated cutoff values is summarized in Table 4. The sensitivity for IgG and IgM was 94.2% and 81.4%, respectively, while the specificity was 87% for IgG and 92.6% for IgM. ELISA IgG and IgM showed 96.6% and 90.4% NPV, suggesting the assay is a very effective assay to rule out the possibility of ST.

**Table 4:** Performance of ELISA using calculated cutoff values from known negative controls at 99% confidence interval^a^

	Scrub typhus positive determined by definition in Table 2	
Parameters	Number or percentage of positives	
ELISA results	IgG	IgM
True positive (TP)	81	70
True negative (TN)	141	150
False positive (FP)	21	12
False negative (FN)	5	16
Sensitivity	94.2	81.4
Specificity	87.0	92.6
Positive predict value (PPV)	79.4	85.4
Negative predict value (NPV)	96.6	90.4
Accuracy	89.5	88.7
Likelihood ratio positive (LR+)	7.27	11.0
Likelihood ratio negative (LR-)	0.07	0.20

^a^Table shows number of samples for true positive, true negative, false positive and false negative. Sensitivity, specificity, PPV, and NPV are shown as percentage. The likelihood ratio was determined by using the following formulas: LR+ = sensitivity/ (1 – specificity), LR- = (1-sensitivity)/specificity. Samples are considered ELISA positive if the OD values are greater than or equal to 0.816 for IgG or 0.320 for IgM

### Evaluation of the performance of ELISA using cutoff values determined based on ROC curve

The distribution of IgG or IgM ELISA OD with scrub typhus positive and negative cases is shown in Additional file 1: Figure S2 with area under curve for IgG and IgM at 95.3% and 91.7%, respectively (Table 5). The maximum J values for IgG and IgM were 0.85 and 0.76, respectively. Consequently, the resulting ELISA cutoff value for IgG was 1.305 and for IgM was 0.3595. The sensitivity for IgG and IgM was 90.7% and 81.4%, respectively, while the specificity was 94.4% for both IgG and IgM. The NPVs for ELISA IgG and IgM were 95.0% and 90.5%, respectively (Table 5).

**Table 5:** Performance of ELISA using cutoff values generated by ROC curves^a^

	Scrub typhus positive determined by definitions in Table 2	
Parameters	Number or percentage of positives	
ELISA results	IgG	IgM
Area under curve (ARC)	95.3	91.7
J (Sensitivity + Specificity – 1)	0.85	0.76
True positive (TP)	78	70
True negative (TN)	153	154
False positive (FP)	9	8
False negative (FN)	8	16
Sensitivity	90.7	81.4
Specificity	94.4	94.4
Positive predict value (PPV)	89.7	88.6
Negative predict value (NPV)	95.0	90.5
Accuracy	93.1	89.9
Likelihood ratio positive (LR+)	16.3	14.7
Likelihood ratio negative (LR-)	0.10	0.20

^a^Table shows number of samples for true positive, true negative, false positive and false negative. AUR, sensitivity, specificity, PPV, and, NPV are shown as percentage. The likelihood ratio was determined by using the following formulas: LR+ = sensitivity/ (1 – specificity), LR- = (1-sensitivity)/specificity. Samples are considered ELISA positive if the OD exceeds or equals to 1.305 for IgG and 0.3595 for IgM

## Discussion

The recombinant protein antigens derived from Kato and Gilliam strains of *Orientia* showed the highest titer of antibodies elicited by corresponding Kato and Gilliam strains while the chimeric C1 protein antigen, derived from Karp and TA763 strains of *Orientia* had highest titer of antibodies elicited in mice by most of the 18 different strains of *Orientia* present in the traditional scrub typhus triangle [25]. Therefore, it is reasonable to assume that the combination of these three antigens result in a broad range reactivity to antibodies elicited by most *Orienti*a species that are important in the endemic areas. This notion is supported by the results of a dot-ELISA assay based on the same three recombinant protein antigens. Rodkvamtook et al. demonstrated that the dot-ELISA assay had very good sensitivity and specificity for ST diagnosis [30]. Our results concluded that the use of these recombinant proteins in ELISA also provided good sensitivity and specificity to diagnose scrub typhus.

The cutoffs were calculated by two different ways. The ROC curve derived cutoffs were generated by comparing the OD values of ELISA and diagnosis of ST using aforementioned ST definition using GraphPad V.5.03. While it provides statistical analysis for the performance of the ELISA, it might not be very useful when the diagnosis could not be made by gold standard IFA or PCR. Furthermore, the need of computer software to calculate the ROC curve derived cutoffs limits the wide usage of this method in rural area. On the contrary, the inclusion of known ST negatives in the ELISA provides an alternative way to calculate cutoffs to determine whether samples are ST positive or negative. Although the diagnosis of ST is best made if a seroconversion or a 4-fold increase in titer between acute and convalescent sera can be documented, it is not very likely for clinicians to wait until the collection of both sera to make the diagnosis and recommend treatment. Consequently, an assay that can accurately diagnose the disease using a single acute serum would be beneficial and clinically relevant. Therefore, we only selected acute sera to be tested by ELISA to evaluate the clinical utility of the ELISA.

A total of 66 positives were diagnosed only by IFA titers of the acute sera (Table 2) while a total of 86 positives were diagnosed by IFA titers and PCR using both acute and convalescent sera (Table 3). Among these additional 20 scrub typhus positives, 17 of them were ELISA positive and 3 were ELISA negatives. All these three negatives were those diagnosed by 4-fold IFA titer increase. These results suggest that ELISA was more sensitive for diagnosing scrub typhus than IFA if only acute sera were tested. This is particularly important as it is not easy to have convalescent sera of patients if their conditions have improved. This may partially explain why 67% (20/30) of patients with only acute sera were ST negatives by IFA but they were ELISA IgG or IgM positive (Additional file 1: Table S2). Among eight patients with co-infection, at least one was ST and leptospirosis co-infection as determined by 4-fold increase of IFA titer for both ST and leptospirosis. This co-infection is known in Thailand [34].

Results in Tables 4 and 5 show that IgG ELISA performed better than IgM regardless what ELISA cutoffs were used. Furthermore, the ELISA assay has similar performance using the ROC curved derived cutoffs in comparison with those using known negative samples. Both IgG and IgM have good LR+ (7.27–16.3) and very low LR- (0.07–0.20), strongly suggest both are very good diagnostic assays [35] even though diagnosis criteria were based on results from both acute and convalescent sera and only acute sera were used for ELISA. Since only the acute specimens from individual patients was used for IgG or IgM ELISA with sensitivity and specificity suitable as diagnostic assays, these results support the clinical utility of either IgG or IgM ELISA to be used for ST diagnosis using only the acute specimens and some known negatives. One limitation of the utility of IgG ELISA as a diagnostic assay is the possibility that it may only apply in this group of Thailand samples since one single IgG titer above 400 was used as one of the criteria to define positive. Nevertheless, the observation of IgG or IgM ELISA showing sufficient sensitivity and specificity suitable for diagnosis is particularly relevant in areas without IFA ability where a screening assay would give clinicians in endemic areas the ability to rule in scrub typhus and thus prescribe doxycycline for prompt treatment [36–38].

The cutoffs generated by ROC curves were all higher than those generated by known negatives using 99% confidence interval. With the higher cutoffs, there would be chances of more false negatives and less false positives, and consequently a decreased sensitivity and a better specificity, this is exactly what we observed. The difficulty with the use of known negatives is associated with the availability of negatives, their average OD values, and the number and types (i.e. healthy individuals and/or patients confirmed scrub typhus negatives but may have other infections) of negatives included. The negatives used in this work included a limited number of healthy individuals and patients of other diseases. Therefore, the performance of the ELISA assay could be further improved by increasing the number and variety of negative samples. It is recommended that a preliminary study be performed using the recombinant proteins-based ELISA with known negatives to establish cutoffs, particularly if IFA can be performed to identify true ST positives. Nevertheless, the cutoffs determined by including known negative controls to calculate using 99% CI is an appropriate approach that could be applied to both IgG and IgM. Recent work by Blacksell et al. [29] evaluated the performance of an InBios IgM ELISA kit using IFA titer or additional criteria as the gold standard demonstrated that the assay performed well, supporting the use of these recombinant proteins in ELISA.

There were quite a few samples (Additional file 1: Table S2) that did not have convalescent sera. Therefore, it was impossible to confirm if they were indeed ST negatives. Furthermore, there was no PCR performed on these samples either. Consequently, they were determined as ST negatives based only on the IFA results of acute sera. Even though these samples were considered negatives by current definition, it is conceivable that they were true positive thus the recombinant-protein based ELISA was actually more sensitive than IFA in detecting the presence of antibodies in acute specimens. This can be supported by the consistent results between ELISA and IFA (Additional file 1: Table S1) at titer 200 and greater, and good agreement with titers less than 200. Alternatively, it is also possible that the level of detectable antibodies in endemic areas was high. Thus the positive ELISA results of 1:100 dilution of patient sera did not necessarily reflect current on-going infection, but rather a background or previous exposure to *Orientia*. Nevertheless, the performance of ELISA could be better if acute and convalescent sera of all patients were used in the evaluation. Recent studies [18, 19] have used LCA to determine the most appropriate IFA cutoff. Though the cutoff values used in this study were not established using LCA, it was determined by previous studies that it could reliably diagnose ST in endemic areas [12]. It is noted that IFA assay is very subjective and different groups may have different cutoffs. This is corroborated by recent publications evaluating the performance of the same commercially available InBios strip for ST diagnosis [12, 26–28] where the performance of the assay was very similar even though the IFA titer used to define ST positive varied by 4-fold. Therefore, one must be cautious when evaluating the performance of an ELISA assay using IFA as the gold standard given the variability of the IFA. Nevertheless, IFA is the current gold standard for the diagnosis of ST and the performance of ELISA was and would be evaluated using IFA data. Consequently, the accuracy of IFA is the intrinsic limitation to determine whether other assays can replace it for diagnosis. This limitation also highlights the importance of a reliable ELISA assay to replace IFA for the accurate diagnosis of ST.

## Conclusions

A recombinant-protein based ELISA for the diagnosis of ST was evaluated using 248 samples of which the diagnosis was made by a combination of IFA titers and PCR results. The ELISA cutoff values determined by ROC curves in general give slightly better but comparable statistical parameters than that calculated by using known negatives and 99% confidence interval. In addition, all statistical parameters support the notion that the combination of these recombinant proteins in ELISA provides a good diagnostic assay for ST. The combination of three-recombinant protein based ELISA assay should be considered as an improved, easy-to-operate and cost effective alternative to the gold standard IFA for acute diagnosis and seroprevalence.

## Additional file

Additional file 1: Table S1. Correlation of IFA titer and ELISA in acute samples. **Table S2.** Distribution of IgG or IgM ELISA positive but diagnosed as scrub typhus negative. **Figure S1.** Correlation of IgG and IgM ELISA OD with single IFA titers at 400. One of the criteria to consider a patient as ST positive was based on IgG or IgM titer > = 400 of a single serum. The ELISA OD of patients was plotted against the IgG or IgM titer <400 (negative, open symbols) or > = 400 (positive, closed symbols). The mean OD of each group was plotted in red. The mean and standard deviation of ELISA OD of IgG (circles) or IgM (triangles). IFA positives (IFA titer > = 400) was significantly different from that of IFA negatives (t test, *p* < 0.0001). **Figure S2.** Correlation of IgG and IgM ELISA OD with ST cases. ST cases were determined by a combination of IFA and PCR results. The ELISA OD of patients who were determined as positives (closed symbols) and negatives (open symbols) were plotted. The mean and standard deviation of ELISA OD of each group was plotted in red. The mean OD of IgG (circles) or IgM (triangles) for determined positives and negatives was significantly different (*t* test, *p* < 0.0001). (DOCX 14 kb)

## Abbreviations

AUC: Area under ROC curve
ELISA: Enzyme linked immunosorbent assay
FN: False negative
FP: False positive
HRP: Horse radish peroxidase
IFA: Indirect immunofluorescent assay
IIP: Indirect immunoperoxidase
LCA: Latent class analysis
LR-: Likelihood ratio negative
LR: Likelihood ratio positive
OD: Optical density
PBS: Phosphate buffered saline
PCR: Polymerase chain reaction
ROC: Receiver operating characteristic
ST: Scrub typhus
STIC: Scrub typhus improved criteria
TN: True negative
TP: True positive
