# Supplementary material for: An ELISA assay using a combination of recombinant proteins from multiple strains of Orientia tsutsugamushi offers an accurate diagnosis for scrub typhus
Source: BMC Infect Dis. 2017 Jun 10;17:413. doi: 10.1186/s12879-017-2512-8 (PMC5466769; doi:10.1186/s12879-017-2512-8)
Supplement: Additional file 1: Table S1. — Correlation of IFA titer and ELISA in acute samples. Table S2. Distribution of IgG or IgM ELISA positive but diagnosed as scrub typhus negative. Figure S1. Correlation of IgG and IgM ELISA OD with single IFA titers at 400. One of the criteria to consider a patient as ST positive was based on IgG or IgM titer > = 400 of a single serum. The ELISA OD of patients was plotted against the IgG or IgM titer <400 (negative, open symbols) or > = 400 (positive, closed symbols). The mean OD of each group was plotted in red. The mean and standard deviation of ELISA OD of IgG (circles) or IgM (triangles). IFA positives (IFA titer > = 400) was significantly different from that of IFA negatives (t test, p < 0.0001). Figure S2. Correlation of IgG and IgM ELISA OD with ST cases. ST cases were determined by a combination of IFA and PCR results. The ELISA OD of patients who were determined as positives (closed symbols) and negatives (open symbols) were plotted. The mean and standard deviation of ELISA OD of each group was plotted in red. The mean OD of IgG (circles) or IgM (triangles) for determined positives and negatives was significantly different (t test, p < 0.0001). (DOCX 14 kb) [file 12879_2017_2512_MOESM1_ESM.docx]

Additional file

## Supplemental Table 1. Correlation of IFA titer and ELISA in acute samples*

|  |  | | | |
| --- | --- | --- | --- | --- |
|  | IgG | | IgM | |
| IFA titer | # of samples | # of ELISA positives | # of samples | # of ELISA positives |
| <1:50 | 170 | 27 | 175 | 15 |
| 1:50 | 9 | 6 | 10 | 6 |
| 1:100 | 9 | 9 | 4 | 2 |
| 1:200 | 8 | 8 | 7 | 7 |
| 1:400 | 19 | 19 | 12 | 12 |
| 1:800 | 5 | 5 | 8 | 8 |
| 1:1600 | 10 | 10 | 14 | 14 |
| 1:3200 | 11 | 11 | 10 | 10 |
| >= 1:6400 | 7 | 7 | 8 | 8 |

## *. ELISA cutoff values used for this comparison were 0.816 for IgG and 0.320 for IgM.

## Supplemental Table 2. Distribution of IgG or IgM ELISA positive but diagnosed as scrub typhus negative*

| Diagnosis | Total # of patients | # of patients without convalescent sera | # of patients with convalescent sera and remained IFA negative |
| --- | --- | --- | --- |
| Unknown | 23 | 18 | 5 |
| leptospirosis | 6 | 2 | 4 |
| Murine typhus | 1 | 0 | 1 |

*. The ELISA cutoff values used for IgG and IgM were 0.816 and 0.320, respectively.

**Supplemental Figure 1. Correlation of IgG and IgM ELISA OD with single IFA titers at 400.** One of the criteria to consider a patient as ST positive was based on IgG or IgM titer >= 400 of a single serum. The ELISA OD of patients with were plotted against the IgG or IgM titer < 400 (negative, open symbols) or >= 400 (positive, closed symbols). The mean OD of each group was plotted in red. The mean and standard deviation of ELISA OD of IgG (circles) or IgM (triangles) IFA positives (IFA titer >=400) was significantly different from that of IFA negatives (t test, p < 0.0001).

**Supplemental Figure 2. Correlation of IgG and IgM ELISA OD with ST cases.** ST cases were determined by a combination of IFA and PCR results. The ELISA OD of patients who were determined as positives (closed symbols) and negatives (open symbols) were plotted. The mean and standard deviation of ELISA OD of each group was plotted in red. The mean OD of IgG (circles) or IgM (triangles) for determined positives and negatives was significantly different (t test, p < 0.0001).
